# Determination of serum CA724 levels using fluorescence immunochromatography

**DOI:** 10.1186/s12896-023-00801-w

**Published:** 2023-08-18

**Authors:** Chang Liu, Cuicui Chen, Fenglan Peng, Huankun Liang, Shuhai Zhong, Tiancai Liu, Laiqing Li, Alexander Boronin, Wenqi Dong

**Affiliations:** 1 Heilongjiang Academy of Chinese Medicine Sciences, Harbin, 150036 China; 2 Guangzhou Youdi Bio-technology Co., Ltd, Guangzhou, 510663 China; 3 Jinan Laide Bio-technology Co., Ltd, Jinan, 271100 China; 4 Taian Disabled Soldiers’ Hospital of Shandong Province, Taian, 271000 China; 5 School of Laboratory Medicine and Biotechnology, Southern Medical University, Guangzhou, 510515 China; 6 Laboratory of Plasmid Biology, G.K. Skryabin Institute Of Biochemistry and Physiology of Microorganisms Russian Academy of Sciences, Pushchino, 142290 Russia; 7 Guangzhou Zhenda Biopharmaceutical Technology Co., Ltd, No. 3 Juquan Road, Huangpu District, Guangzhou, 510663 China

**Keywords:** CA724, Biomarker, Gastric cancer, Time-resolved fluorescence immunochromatography, Eu

## Abstract

**Background:**

Carbohydrate antigen 724 (CA724) is a sensitive and specific indicator for multiple malignant tumors. The aim of this study was to establish a Eu-time resolved fluorescence immunochromatography (Eu-TRFICO) method for quantitative detection of CA724 in serum.

**Methods:**

Eu-TRFICO strips were optimized and assembled. The sensitivity, specificity and precision were evaluated using CA724 standard dilutions and matrix serum. Meanwhile, the reference interval, comparison, and sensitivity/specificity were performed using clinical negative/positive gastric cancer serum samples.

**Results:**

The standard curve equation was y = 9.869 x − 154.12 (*R*^*2*^ = 0.993), and the sensitivity was 0.42 U/mL. The common interferents in serum could not affect the quantitative results with low cross-reactivities (all no more than 1.09%). All average recoveries of the intra- and interbatch ranged from 102.38 to 106.40%, and all *CVs* were below 10%. The reference interval of the healthy subjects was < 4.68 U/mL and the reference interval of the subjects with grade I/II gastric cancer was > 9.54 U/mL. Additionally, a high Pearson *r* (0.9503) and sensitivity/specificity (92.86%/94.20%) were obtained.

**Conclusion:**

This study prepared Eu-TRFICO strips with high sensitivity, specificity, precision and satisfactory clinical testing performance, which provides more options for clinical quantitative and convenient testing of CA724.

## Background

CA724 has been identified as a tumor-associated glycoprotein-72 antigen (TAG-72) for more than 40 years and is considered a promising marker in oncology. Numerous studies have outlined that CA724 is an independent marker that can be used for the prognosis and assessment of recurrences in gastrointestinal malignancies, lung, ovarian, endometrial, breast cancer, etc. [1–3]. In particular, elevated serum levels of CA724 have been observed in a significant proportion of patients with gastric cancer (GC), and higher serum CA724 levels are positively related to TNM stage, distant metastasis, recurrence, and poor overall survival [4, 5]. A meta-analysis suggested that the testing of serum CA724 could be acceptable for the diagnosis of GC, particularly among Chinese individuals, and higher levels of CA724 concentrations predict higher mortality rates due to GC [6, 7]. Therefore, the quantitative detection of serum CA724 has significant clinical value for various malignant tumors, especially GC.

Currently, the clinical detection method for serum CA724 is mainly chemiluminescence immunoassay (CLIA). There is an urgent need for fast and simple detection methods in clinical practice. Considering that the traditional gold nanoparticle-based strip assay (GNP-SA) is very fast and convenient but has lower sensitivity, time-resolved fluorescence immunochromatography (TRFICO) was developed recently with increasing assay sensitivity [8]. Hence, we aimed to establish Eu-TRFICO for CA724 quantitative detection in serum. In this study, we first optimized and assembled Eu-TRFICO strips. After adding serum samples and reacting for 15 min, the fluorescence value was detected by a fluorescence detector to calculate the serum CA724 levels. Additionally, we evaluated the sensitivity, specificity, precision, reference interval and clinical sample comparison testing and found that the Eu-TRFICO strips met clinical testing requirements.

## Results

### Standard curves and sensitivity

**Fig. 1:**
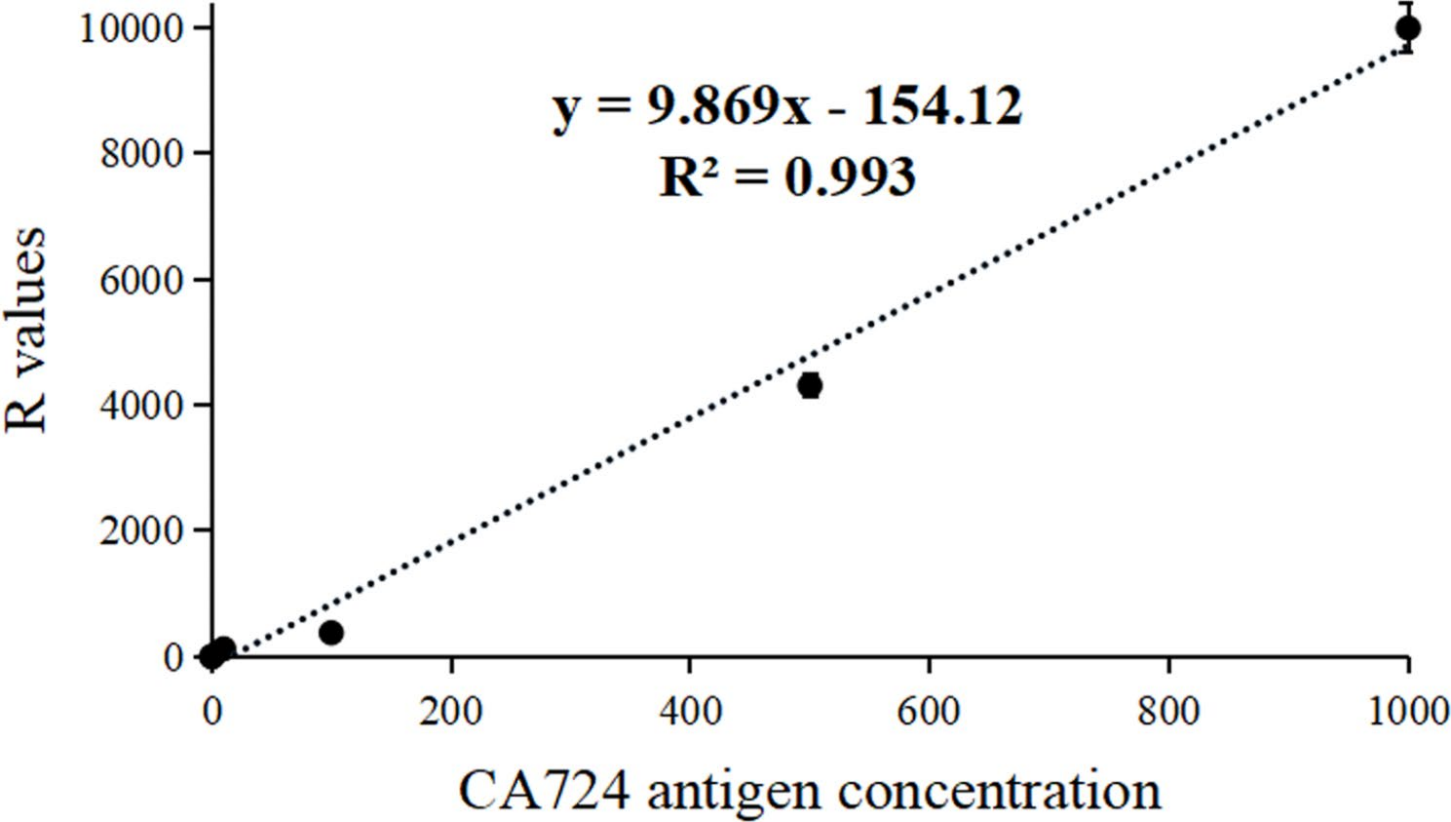
The standard curve of these Eu-TRFICO strips for CA724 quantitative detection. R = H_T_/H_C_.

The standard curve of Eu-TRFICO strips for CA724 quantitative detection is presented in Fig. 1. The standard curve equation was y = 9.869 x − 154.12 (*R*^*2*^ = 0.993). Wide-range (0.1–1000 U/mL) CA724 concentrations and R values (fluorescence values) exhibited a good linear relationship. Based on this standard curve equation, we calculated that the analytical sensitivity of these Eu-TRFICO strips for CA724 was 0.42 U/mL.

### Specificity results

Eu-TRFICO strips detected high concentrations of 8 interferents (SA, hemoglobin, bilirubin, cholesterol, CEA, CA19-9, IL-6 and TNF-α) that were added to the control serum, and the results are shown in Table 1. There was very low cross-reactivity (all no more than 1.09%), indicating that these Eu-TRFICO strips had high specificity for CA724, and the common interferents in serum could not affect the quantitative results.

**Table 1:** Specificity results of the Eu-TRFICO strips

Interferents (500 ng/mL)	Determined concentrations	Basal concentration	Cross-reactivity (%)
SA	7.87 ± 0.74	3.56 ± 0.31	0.86
Hemoglobin	8.80 ± 0.82		1.05
Bilirubin	7.67 ± 0.55		0.82
Cholesterol	9.00 ± 0.62		1.09
CEA	7.16 ± 0.31		0.72
CA19-9	7.16 ± 0.45		0.72
IL-6	8.27 ± 0.29		0.94
TNF-α	8.87 ± 0.38		1.06

### Precision results

The precision of the Eu-TRFICO strips was assessed by detecting the control serum that had been spiked with CA724 (5, 50 and 500 U/mL) standards. As shown in Table 2, all average recoveries of the intra- and interbatch ranged from 102.38 to 106.40%, and all *CVs* were below 10%. The precision results indicated that the precision of the Eu-TRFICO strips was high enough to meet the requirements of clinical immunoassays.

**Table 2:** Precision results of the Eu-TRFICO strips

	Spiked concentration	mean ± *SD*	Recovery (%)	*CV* (%)
Intrabatch (n = 5)	5	8.75 ± 0.52	103.80	5.94
	50	55.42 ± 2.53	103.72	4.57
	500	515.48 ± 18.54	102.38	3.60
Interbatch (n = 5)	5	8.88 ± 0.63	106.40	7.09
	50	56.65 ± 3.25	106.18	5.74
	500	520.17 ± 21.36	103.32	4.11

### Reference intervals

The concentration values of CA724 in different populations were in accordance with the normality distribution, so the normal distribution method was used to calculate the reference interval. For the healthy subjects, the cutoff value was 4.68 U/mL, so its reference interval was < 4.68 U/mL. For the subjects with grade I/II gastric cancer, the cutoff value was 9.54 U/mL, so its reference interval was > 9.54 U/mL (Table 3).

**Table 3:** The reference intervals of healthy subjects and grade I/II gastric cancer subjects by Eu-TRFICO strips

Subjects	Mean	*SD*	Formula	Cutoff (U/mL)
Healthy subjects	3.32	0.83	mean + 1.64*SD*	4.68
I/II grade gastric cancer	13.07	2.15	mean − 1.64*SD*	9.54

### Comparison with the registered kit

**Fig. 2:**
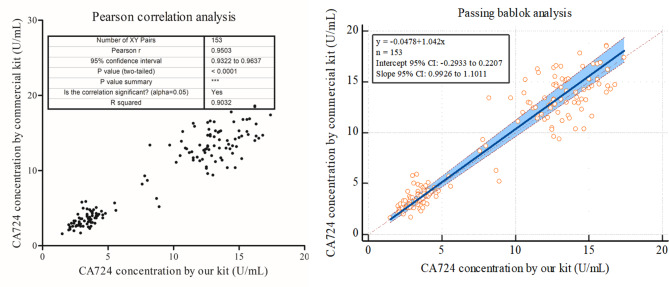
Pearson correlation analysis and Passing bablok analysis of CA724 levels between Eu-TRFICO strips and commercial kits

The concentrations of CA724 in 69 healthy normal subjects and 84 subjects with grade I/II gastric cancer were statistically analyzed/graphed using GraphPad Prism 5 and MedCalc software. The comparison results are shown in Fig. 2. For Pearson correlation analysis: Pearson *r* was 0.9503. For Passing bablok analysis: The regression equation is y = -0.0478 + 1.042x, the intercept 95% confidence interval (CI): -0.2933 to 0.2207, and the slope 95% CI: 0.9926 to 1.1011. Pearson correlation analysis and Passing bablok analysis indicated that the measurement results of Eu-TRFICO strips and commercial kit have good consistency. In accordance with the reference interval, the negative/positive results were determined, the numbers of true positives, false-negatives, true negatives and false-positives were counted, and then the sensitivity and specificity were calculated. The sensitivity was 92.86%, and the specificity was 94.20%. These results showed that these Eu-TRFICO strips have acceptable clinical testing performance.

## Discussion

TRFICO is an important new method that has been applied to the quantitative detection of multiple samples, including in the food industry and human clinics [8–10]. In 2022, Chen et al. established a highly sensitive time-resolved fluorescence microsphere lateral flow immunochromatographic assay (TRFM-LFIA) for the small molecule compound propiconazole [11]. In 2018, Wang et al. developed a portable TRFICO for the sensitive and on-site determination of CA19-9 in serum [12]. You can think of it this way, TRFICO is an improvement of GNP-SA. As the fluorescence lifetime of lanthanide chelates is several orders of magnitude longer than that of the background interference fluorescence, it is not easily affected by sample autofluorescence, and TRFICO collects only the fluorescence signal of the sample by the time delay technique [13, 14]. Therefore, the detection sensitivity and reliability are greatly improved in TRFICO. In summary, TRFICO has many advantages: fast and simple operation (approximately 15 min), high accuracy and reproducibility, high stability and practicality for on-site use. However, TRFICO requires a fluorescence detector, and the expensive detector limits its widespread application [15]. Recently, the emergence of portable TRFICO readers has promoted its rapid, widespread use and application [16]. In this study, we established a Eu-TRFICO method for CA724 quantitative detection based on a portable fluorescence reader.

Currently, there is no Eu-TRFICO method for CA724 quantitative detection. In this study, we attempted to fully leverage the advantages of immunochromatography and time-resolved immunoassay to establish a Eu-TRFICO method. First, Eu-fluorescent microspheres were combined with anti-CA724 antibodies to prepare Eu-fluorescent microsphere-anti-CA724 antibody conjugates. After the conjugates fully reacted with the CA724 antigen in the sample, Eu-fluorescent microsphere-anti-CA724 antibody-CA724 antigen conjugates were captured on the T-line that was coated with the anti-CA724 paired antibodies. To improve the accuracy of testing, we used the independent C-line color rendering method, and Eu-fluorescent microsphere-DNP-BSA conjugates were captured on the C-line that was coated with anti-DNP antibodies [17]. Finally, the portable fluorescence reader read the fluorescence intensity of the T-line and C-line and then calculated the CA724 concentration according to the standard curve. The prepared Eu-TRFICO strips showed high sensitivity (0.42 U/mL) with low cross-reactivities, all average recoveries of the intra- and interbatch ranged from 102.38 to 106.40%, and all CVs were below 10%. Compared with the sensitivity of reported methods (ELISA: 0.31 U/mL, radioimmunoassay: 0.8 U/mL, time-resolved immunofluorometric assay: 0.55 U/mL, LFIA 0.38 U/mL) [18–21], the Eu-TRFICO method (0.42 U/mL) exhibited a comparable or superior detection performance with the additional advantages of the detection time (15 min), linearity range (0.1–1000 U/mL), and *CV* (all below 10%).

As a biomarker, CA724 can achieve improved sensitivity and specificity when selecting suitable reference values, improving detection techniques, and identifying the risk threshold [22]. A study on individuals in the Chinese region found that CA724 reference values show spatial autocorrelation and regional variation, and geographical environmental factors (sunshine, temperature, humidity, etc.) affected healthy Chinese adult CA724 reference values [23]. The reference interval of these TRFICO strips was obtained from 69 healthy normal subjects and 84 subjects with grade I/II gastric cancer, and the preliminary reference interval was < 4.68 U/mL for healthy subjects, and > 9.54 U/mL for the subjects with grade I/II gastric cancer, which means that when CA724 > 9.54 U/mL, the patients are more likely to suffer from GC (this is not the only diagnostic criterion but should also be combined with clinical symptoms and other testing methods). Under the current conditions, the Pearson correlation *r* between these Eu-TRFICO strips and the commercial kits reached 0.9503, the sensitivity was 92.86%, and the specificity was 94.20%. Attentively, this reference interval is preliminary and variable and is influenced by the geographical detection area, samples, test steps, etc.

In conclusion, a rapid, sensitive Eu-TRFICO method based on a portable fluorescence reader was first established and validated for the quantitative detection of CA724 in serum. The preparation of Eu-TRFICO strips can provide an efficient and useful technical choice for the rapid quantitative detection of CA724 in serum. Numerous studies have confirmed that detecting CA724 alone is of no value in predicting gastric cancer, and combined testing is valuable [24, 25]. Therefore, this study is only a preliminary exploration of the Eu-TRFICO methodology and is the first stage of the entire study, and the Eu-TRFICO method may still require further optimization and improvement. Ultimately, our goal is to develop a series of biomarker detection TRFICO methods, which can be combined to achieve accurate and rapid prediction of GC staging, metastasis, recurrence, etc.

## Materials and methods

### Antigen, antibody, reagents and clinical samples

CA724 recombinant antigen and anti-CA724 paired antibodies (coating and detection antibodies) were obtained from Yidenuo Biotechnology (Guangzhou, China). Eu-time resolved fluorescent microspheres (particle size 210 nm) were purchased from Microdetection (MD018, China). Polyvinyl chloride (PVC) soleplate, absorbent paper, sample pad and bonding pad were purchased from Sartorius. 1-(3-Dimethylaminyl)-3-ethylenediamine hydrochloride (EDC) and N-hydroxysuccinimide (NHS) were purchased from Sigma (St Louis, MO, USA). Dinitrophenol (DNP) antigen (DNP-BSA) (EDD0405A) and its DNP monoclonal antibody (EKY0817A) were purchased from Seebio.

The serum samples came from Taian Disabled Soldiers’ Hospital of Shandong Province, including healthy control subjects (M/F 45/101; age range 34–72 years) and patients with gastric cancer (I and II grade) (M/F 39/45; age range 41–74 years). All subjects were diagnosed through gastroscopy, biopsy of the gastric antrum/gastric mucosa and clinical examination.

### Conjugation of anti-CA724 antibodies to fluorescent microspheres

The Eu-time resolved fluorescent microspheres were activated by the classical EDC/NHS method and then coupled with anti-CA724 antibodies. After the Eu-time resolved fluorescent microspheres were washed with 50 mM morpholine sulfonic acid buffer (pH 6.0), 14 µL 1% EDC and 132 µL 1% NHS were added and activated for 30 min at room temperature by shaking. After centrifugation at 4 °C and 15,000 rpm/min, 1.1 mg anti-CA724 antibodies was added to the activated fluorescent microspheres (1 mL) and gently shaken for 2 h at room temperature. The microspheres were washed (25 mmol/L Tris-base + 0.2% Tween 20 + 0.15 mol/L NaCl + 0.05% ProClin 300, pH 7.8) and blocked (50 mmol/L PB + 5% BSA, pH 8.0), resuspended in preservation buffer (25 mmol/L Tris-base + 0.05% Tween 20 + 0.15 mol/L NaCl + 0.05% ProClin 300 + 1%BSA + 5% trehalose, pH 7.2), and stored in the dark at 4℃.

### Conjugation of DNP-BSA to fluorescent microspheres

Conjugation of DNP-BSA to fluorescent microspheres was similar to anti-CA724 antibody conjugation. The ratio was 0.6 mg DNP-BSA:1 mL fluorescent microspheres.

### Coating of NC membrane

Coating of the NC membrane was also performed using the XYZ3060 3D spraying platform. In this study, we selected Millipore’s nitrocellulose (NC) membrane (HF13502XSS) as the reaction carrier for immunochromatographic reagent strips. The NC membrane has a detection area (T-line) and a quality control area (C-line). Anti-CA724 paired antibodies and DNP monoclonal antibodies were coated on the T and C lines, respectively. The spraying speed parameter was set to 0.8 µL/cm. NC membranes were dried in a 37 °C air drying oven for 2 h and stored in a moisture-proof cabinet.

### Pretreatment of sample pads and conjugate pads

Sample pad pretreatment: Cut the sample pads into 17 mm (width)×300 mm (length) pieces and soak them in the sample pad pretreatment buffer (10 mmol/L sodium tetraboric acid, 1% polyvinyl pyrrolidone (PVP), 0.2% sodium casein, 1% Trition-X100, 1% S9, 0.05% ProClin 300) for 1.5 h. After draining, place them in a 37 °C air drying oven for 3 h and store in a moisture proof cabinet for future use. Conjugate pads Pretreatment: Cut the conjugate pads into 10 mm (width)× 300 mm (length) sizes and soak them in the bonding pad pretreatment buffer (50 mmol/L Na_2_HPO_4_12H_2_O, 0.5% PVA, 0.5% BSA, l%Trition-X100, pH 7.4) for 1 h. After draining, place them in a 37 °C air drying oven for 3 h and store in a moisture proof cabinet for future use.

### Solidification of anti-CA724 antibody-fluorescent microspheres

Anti-CA724 antibody-fluorescent microsphere conjugates and DNP-BSA-fluorescent microsphere conjugates were diluted to concentrations of 1.2 mg/mL and 1.0 mg/mL, respectively, and thoroughly mixed at a ratio of 1:1. The XYZ3060 3D spraying platform (Biodot, USA) was used for evenly spraying the bonding pad. The spraying speed parameter was set to 10 µL/cm. The conjugate pads were dried in a 37 °C air drying oven for 2 h and stored in a moisture-proof cabinet.

### Assembly of reagent strips

The reagent strips in this study consist of 5 parts: PVC soleplate, NC membrane, absorbent paper, sample pad, and bonding pad. The sample pad, bonding pad, NC membrane, and absorbent paper are sequentially fixed on the PVC board. After assembly, the PVC board was cut into 3 mm wide strips using a slitting machine, loaded into a plastic cartridge, and stored in a moisture-proof cabinet for future use.

### Test procedure

**Fig. 3:**
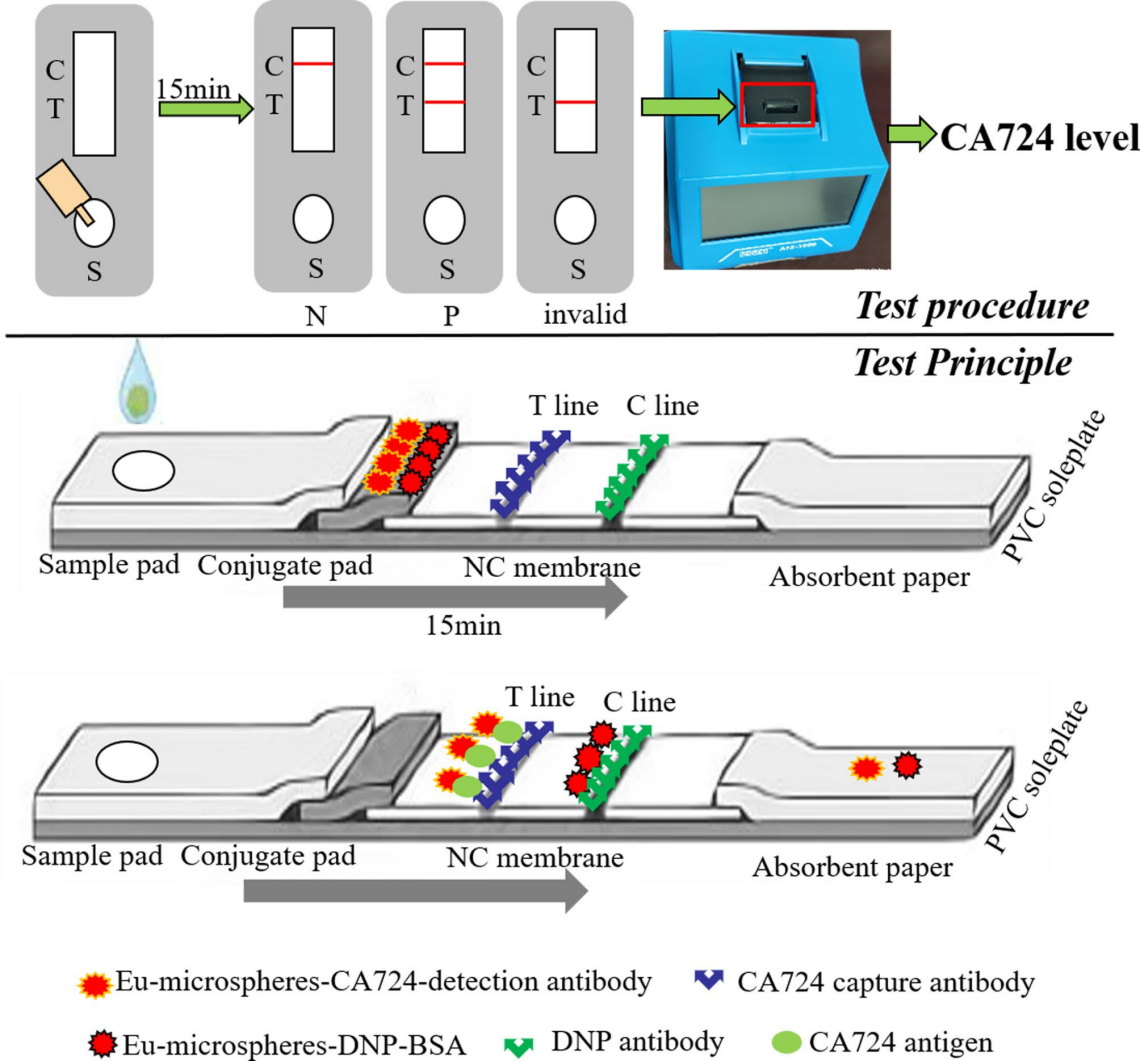
The test procedure and test principle of CA724 by these Eu-TRFICO strips

The reaction mode of these strips is the double antibody sandwich method, and the test procedure and test principle are shown in Fig. 3. The specific steps were as follows: Dilute the serum sample or standards at a dilution of 1:20, shake and mix well. The strip was placed horizontally, and 80 µL of the above suspension was dropped onto the detection well and incubated for 15 min at room temperature. Then, the strip was placed into a time-resolved fluorescence immunochromatographic test detector (Microdetection, MD-200, China). H_T_ (T line fluorescence value), H_c_ (C line fluorescence value) and their ratio R (H_T_/H_c_) were recorded, and the CA724 level in serum was analyzed using a standard curve.

### Standard curve and sensitivity assay

The standards of CA724 were diluted to 0, 0.1, 1, 10, 100, 500 and 1000 U/mL and then detected by our strips. The concentration values of CA724 standards were plotted on the X-axis, and the corresponding R (H_T_/H_c_) values were plotted on the Y-axis. A linear fit was performed, and a standard curve was drawn. U/mL dilutions as samples were detected 10 times, and 10 CA724 concentration values were obtained using a standard curve. Then, the mean and standard deviation (*SD*) were calculated, and the sensitivity = mean + 2*SD*.

### Specificity assay

Serum albumin (SA), hemoglobin, bilirubin, cholesterol, carcinoembryonic antigen (CEA), carbohydrate antigen 19 − 9 (CA19-9), interleukin 6 (IL-6) and tumor necrosis factor-α (TNF-α), which are the common components in a serum [26], were selected as interferents for the specificity assay. High concentrations of interferents (500 ng/mL) were added to the control serum and detected using our strips. The levels of these interferents in control serum (basal concentration) were also detected using our strips. Cross-reactivities (%) = (determined concentration-basal concentration)/interferent concentrations×100%.

### Precision assay

Three concentration levels (high, medium, low) of standard dilutions were added to the control serum, and their fluorescence values were measured. The CA724 concentration and the *SD*s were calculated using three batches of strips. Recovery (%) = (determined concentration-basal concentration)/spiked concentration×100%. *CV*(%) = SD/mean×100%.

### Reference interval

A total of 69 healthy normal subjects and 84 subjects with grade I/II gastric cancer were used for reference interval determination. These strips were used to detect and calculate the CA724 levels. The CA724 concentration values were subjected to a normality test using SPSS 20.0, and the one-sided upper/lower limit of the 95% reference interval range was applied to identify the reference intervals. The cutoff values were calculated using the following formula: cut off = mean − 1.64*SD* (one-sided lower limit), and cut off = mean ± 1.64*SD* (one-sided upper limit) [27].

### Comparison with the registered kit

The sera of 69 healthy normal subjects and 84 subjects with grade I/II gastric cancer were used for comparison. The serum samples were simultaneously measured by these time-resolved fluorescence immunochromatographic strips and the registered CA724 electrochemiluminescence kit (Roche, Registration Certificate For Medical Device: 20,183,402,635, Germany). Pearson correlation analysis and Passing bablok analysis were performed using GraphPad Prism 5 and MedCalc software. Meanwhile, the sensitivity ((true positive/(true positive + false-negative) × 100%) and specificity ((true negative/(true negative + false-positive) × 100%) of the two methods were calculated.

### Statistical analysis

Data were statistically analyzed and graphed using GraphPad Prism 5 (GraphPad Software, USA). All results are presented as the mean ± *SD*.

## Data Availability

All data generated or analysed during this study have been provided in a supplementary file, and can be also available from the authors upon reasonable request.

## Abbreviations

CA724: Carbohydrate antigen 724
Eu-TRFICO: Eu-time resolved fluorescence immunochromatography
GC: Gastric cancer
CLIA: Chemiluminescence immunoassay
GNP-SA: Gold nanoparticle-based strip assay
PVC: Polyvinyl chloride, NC:nitrocellulose
EDC: 1-(3-dimethylaminyl)-3-ethylenediamine hydrochloride
NHS: N- Hydroxysuccinimide
DNP: Dinitrophenol
PVP: Polyvinyl pyrrolidone
SD: Standard deviation
SA: Serum albumin
CEA: Carcinoembryonic antigen
CA19-9: Carbohydrate antigen19-9
IL-6: Interleukin 6
TNF-α: Tumor necrosis factor-α
